# Supplementary material for: Host Barriers Limit Viral Spread in a Spillover Host: A Study of Deformed Wing Virus in the Bumblebee Bombus terrestris
Source: Viruses. 2024 Apr 15;16(4):607. doi: 10.3390/v16040607 (PMC11053533; doi:10.3390/v16040607)
Supplement: Supplementary file 1 [file viruses-16-00607-s001.zip › Supplementary material Viruses MDPI Streicher et al..pdf]

Supplementary material to:

Title: **Host barriers limit viral spread in a spillover host: a study of deformed wing virus in the bumble bee *Bombus terrestris***

Authors: Tabea Streicher[1], Pina Brinker[1,2], Simon Tragust[1], and Robert J. Paxton[1,2]

[1] General Zoology, Institute for Biology, Martin Luther University Halle-Wittenberg, Hoher Weg 8, 06120 Halle (Saale), Germany

[2] German Centre for Integrative Biodiversity Research (iDiv) Halle-Jena-Leipzig, Puschstraße 4, 04103 Leipzig, Germany

Submitted to: *Viruses* MDPI

Content:

- **Table S1** Colony screening results for six common honey bee viruses
- **Table S2** Raw data on samples and PCR results underpinning Figures 1, 2 and 3 (main manuscript).
- **Table S3** Results of capillary electrophoresis to identify one-step RT-PCR products of haemolymph from bumble bees and honey bees fed DWV-B
- **Figure S1** Electropherograms of one-step RT-PCR products of haemolymph from bumble bees and honey bees fed DWV-B
- **Table S4** Screening pollen viruses

Table S1 Colony screening results of experimental *A. mellifera* and *B. terrestris* colonies for six common honey bee viruses, undertaken by real-time PCR. Samples were run in technical duplicates. N/A = negative. Honey bee beta-actin served as a quality control internal 'reference' for RNA extraction and cDNA synthesis.

Colony	Actin	DWV-A	DWV-B	BQCV	SBV	SBPV	CBPV
A. mellifera	27.26	N/A	37.35	39.35	N/A	N/A	N/A
R25	-	N/A	N/A	37.79	N/A	N/A	N/A
B. terrestris	23.46	N/A	N/A	N/A	N/A	N/A	N/A
Colony 1	23.04	N/A	N/A	N/A	N/A	N/A	N/A
B. terrestris	23.38	N/A	N/A	N/A	N/A	N/A	N/A
Colony 2	23.17	N/A	N/A	N/A	N/A	N/A	N/A
B. terrestris	22.78	N/A	N/A	N/A	N/A	N/A	N/A
Colony 3	22.66	N/A	N/A	N/A	N/A	N/A	N/A
B. terrestris	23.59	N/A	N/A	N/A	N/A	N/A	N/A
Colony 4	23.49	N/A	N/A	N/A	N/A	N/A	N/A
B. terrestris	23.48	N/A	N/A	N/A	N/A	N/A	N/A
Colony 5	23.50	N/A	N/A	N/A	N/A	N/A	N/A
B. terrestris	19.42	N/A	N/A	N/A	N/A	N/A	N/A
Colony 6	19.53	N/A	N/A	N/A	N/A	N/A	N/A
B. terrestris	21.59	N/A	N/A	N/A	N/A	N/A	N/A
Colony 7	21.47	N/A	N/A	N/A	N/A	N/A	N/A
B. terrestris	24.67	N/A	N/A	N/A	N/A	N/A	N/A
Colony 8	24.31	N/A	N/A	N/A	N/A	N/A	N/A

Table S2 Raw data on samples and PCR results underpinning Figures 1, 2 and 3 (main manuscript).

The Excel file is available at: link article doi

Table S3 Results of capillary electrophoresis of PCR products using haemolymph as template from DWV-B-fed or control-fed *B. terrestris* and *A. mellifera* workers. Alignment marker: 15 bp/3 kb; size marker: 50-800 bp; expected PCR product length of DWV-B: 140 bp. * random PCR product with a melt temperature of 74.0°C that was present in all hemolymph samples tested for DWV-B. Figure S1 provides visualisation of these results. (Positions A2-A5; A10-A11 belong to a different data set and are not shown here).

Overall Result Table<br>C220802A22_2023-06-09_12-24-30											
A1<br>Sizemarker		A6<br>Control fed *Bombus*		A7<br>Control fed *Bombus*		A8<br>Control fed *Apis*		A9<br>DWV-B fed *Apis*		A12<br>DWV-B fed *Bombus*	
Size [bp]	Conc. [ng/µl]	Size [bp]	Conc. [ng/µl]	Size [bp]	Conc. [ng/µl]	Size [bp]	Conc. [ng/µl]	Size [bp]	Conc. [ng/µl]	Size [bp]	Conc. [ng/µl]
15	n/a	15	n/a	15	n/a	15	n/a	15	n/a	15	n/a
50	0.33	57*	0.59	57*	0.66	57*	0.8	56*	0.19	56*	0.36
95	0.33	3000	n/a	3000	n/a	3000	n/a	**148**	1.27	741	0.09
143	0.34							3000	n/a	3000	n/a
200	0.34										
254	0.35										
305	0.83										
407	0.33										
507	0.34										
596	0.34										
684	0.38										
766	0.31										
3000	n/a										

Sample Header			
Position:	A6	Run Date:	09/06/2023 12:24:30
Sample Info:			
Plate ID:	C220802A22_2023-06-09_12-24-30, R: 1, E: 1		

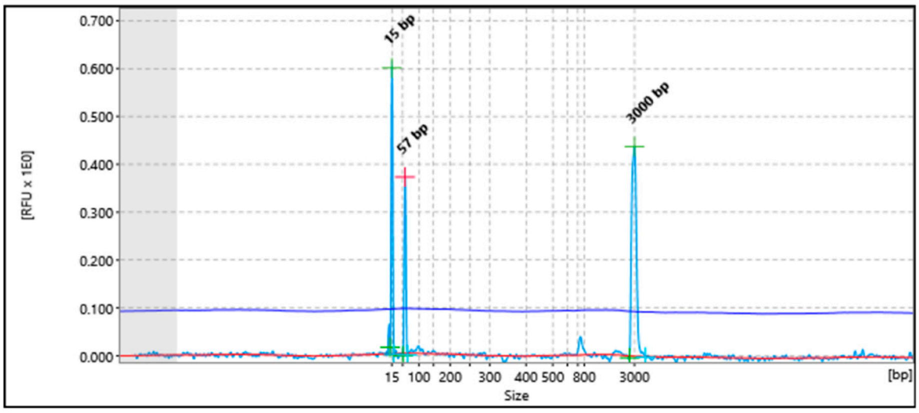

Fig. S1a Haemolymph of a control-fed *B. terrestris* worker (lane A6)

Sample Header			
Position:	A8	Run Date:	09/06/2023 12:24:30
Sample Info:			
Plate ID:	C220802A22_2023-06-09_12-24-30, R: 1, E: 1		

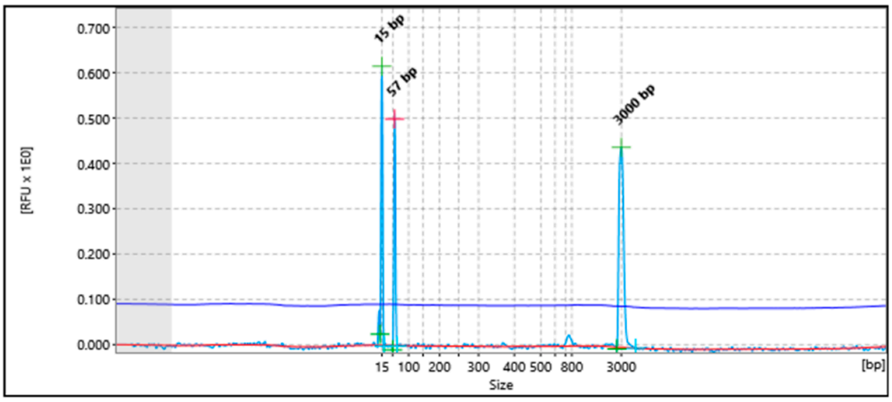

Fig. S1b Haemolymph of a control-fed *A. mellifera* worker (lane A8)

Sample Header			
Position:	A12	Run Date:	09/06/2023 12:24:30
Sample Info:			
Plate ID:	C220802A22_2023-06-09_12-24-30, R: 1, E: 1		

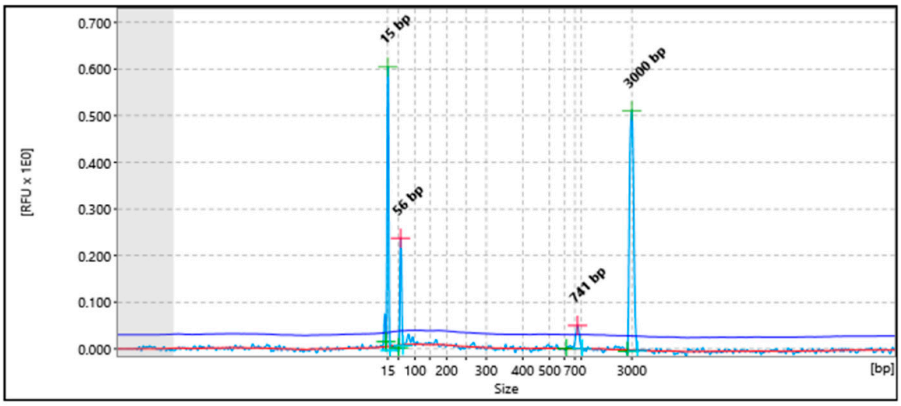

Fig. S1c Haemolymph of a DWV-B-fed *B. terrestris* worker (lane A12)

Sample Header			
Position:	A9	Run Date:	09/06/2023 12:24:30
Sample Info:			
Plate ID:	C220802A22_2023-06-09_12-24-30, R: 1, E: 1		

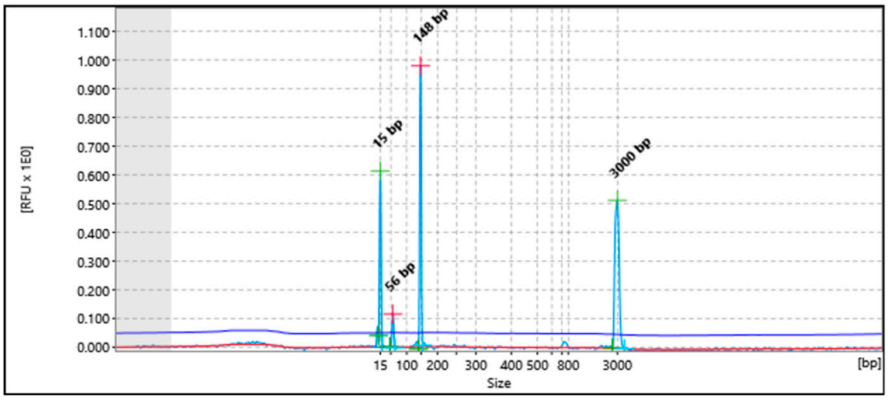

Fig. S1d Haemolymph of a DWV-B-fed *A. mellifera* worker (lane A9)

Figure S1 Electropherograms of PCR products using haemolymph as template, collected from control-fed *B. terrestris* **(1a)**, and *A. mellifera* **(1b)** workers and DWV-B-fed *B. terrestris* **(1c)** and *A. mellifera* **(1d)** workers (data presented in Table S3). All samples show a PCR product with a length of 56-57 bp when tested for the presence of DWV-B, likely due to primer dimerization, indicated through the short fragment length. DWV-B was detected only in DWV-B-fed *A. mellifera* (**1d**: a product length of 148 bp), while no DWV-B was detected in the hemolymph of a DWV-B-fed *B. terrestris* (**1c**).

Table S4 Results (Cq value) of honey bee pollen screening for DWV-A, DWV-B and BQCV by RT-PCR. Commercially available honey bee pollen regularly carries bee viruses. Shown are two different pollen batches; gamma irradiated pollen from Great Britain and non-irradiated pollen from Austria. The latter was used to feed *Bombus terrestris* colonies in the laboratory as a source of bumble bees for our experiments.

**Pollen sample**	**DWV-A**	**DWV-B**	**BQCV**
**gamma irradiated**			
pollen sample a	N/A	N/A	N/A
(technical duplicate of a)	N/A	N/A	N/A
pollen sample b	N/A	N/A	N/A
(technical duplicate of b)	N/A	N/A	N/A
pollen sample c	N/A	39.10	38.02
(technical duplicate of c)	N/A	37.93	38.31
pollen sample d	N/A	N/A	N/A
(technical duplicate of d)	N/A	N/A	N/A
**non-irradiated pollen**			
pollen sample 1	37.01	N/A	23.78
pollen sample 2	37.02	N/A	28.75
pollen sample 3	39.20	N/A	30.04
pollen sample 4	37.13	38.62	30.04
